# Prevention of the foreign body response to implantable medical devices by inflammasome inhibition

**DOI:** 10.1073/pnas.2115857119

**Published:** 2022-03-17

**Authors:** Damiano G. Barone, Alejandro Carnicer-Lombarte, Panagiotis Tourlomousis, Russell S. Hamilton, Malwina Prater, Alexandra L. Rutz, Ivan B. Dimov, George G. Malliaras, Stephanie P. Lacour, Avril A. B. Robertson, Kristian Franze, James W. Fawcett, Clare E. Bryant

**Affiliations:** ^a^John van Geest Centre for Brain Repair, Department of Clinical Neurosciences, University of Cambridge, Cambridge CB2 0PY, United Kingdom;; ^b^Division of Neurosurgery, Department of Clinical Neurosciences, University of Cambridge, Cambridge CB2 0PY, United Kingdom;; ^c^Department of Veterinary Medicine, University of Cambridge, Cambridge CB3 0ES, United Kingdom;; ^d^Electrical Engineering Division, Department of Engineering, University of Cambridge, Cambridge CB3 0FA, United Kingdom;; ^e^Department of Physiology, Development and Neuroscience, University of Cambridge, Cambridge CB2 3DY, United Kingdom;; ^f^Centre for Trophoblast Research, University of Cambridge, Cambridge CB2 3EG, United Kingdom;; ^g^Department of Genetics, University of Cambridge, Cambridge CB2 3EH, United Kingdom;; ^h^Bertarelli Foundation Chair in Neuroprosthetic Technology, Laboratory for Soft Bioelectronics Interface, Institute of Microengineering, Institute of Bioengineering, Centre for Neuroprosthetics, Ecole Polytechnique Fédérale de Lausanne, 1202 Geneva, Switzerland;; ^i^School of Chemistry and Molecular Biosciences, The University of Queensland, St Lucia, QLD 4072, Australia;; ^j^Max-Planck-Zentrum für Physik und Medizin, 91054 Erlangen, Germany;; ^k^Institute of Medical Physics and Microtissue Engineering, Friedrich-Alexander University Erlangen–Nuremberg, 91052 Erlangen, Germany;; ^l^Centre for Reconstructive Neuroscience, Institute for Experimental Medicine, Czech Academy of Sciences, 142 20 Prague 4, Czech Republic;; ^m^Division of Medicine, University of Cambridge, Cambridge CB2 0PY, United Kingdom

**Keywords:** foreign body reaction, NLRP3 inflammasome, neural interfaces, MCC950

## Abstract

Fibrotic scarring secondary to the foreign body reaction (FBR) generates a physical barrier obstructing the functional interaction of implantable medical devices with the host tissue. The mechanistic basis of the FBR is poorly understood, restricting the current therapeutic options to prevent it. Here, we show that in a peripheral nerve injury-implant model (NI) the FBR has a dysregulated innate immune profile recruiting M1-like activated macrophages, immature macrophages, activated dendritic cells, and immature dendritic cells compared with nerve injury alone, which recruits predominantly M2-like macrophages. The gene signature of the FBR shows increased myofibroblast activity, explaining why collagen and scarring are present, but also up-regulation of inflammasome constituents. Local delivery of the nucleotide-binding oligomerization domain-like receptor family pyrin domain containing 3 (NLRP3) inflammasome inhibitor MCC950, through its incorporation into the silicone coating of implants, reduced the inflammation and fibrosis associated with both NI and subcutaneous implantable devices. In the NI model, MCC950 did not affect neuronal repair. Inhibition of the NLRP3 inflammasome may, therefore, be a promising therapeutic approach to prevent the FBR, hence prolonging the functional lifespan of implantable medical devices and neural implants.

## Significance

Implantable electronic medical devices (IEMDs) are used for some clinical applications, representing an exciting prospect for the transformative treatment of intractable conditions such Parkinson’s disease, deafness, and paralysis. The use of IEMDs is limited at the moment because, over time, a foreign body reaction (FBR) develops at the device–neural interface such that ultimately the IEMD fails and needs to be removed. Here, we show that macrophage nucleotide-binding oligomerization domain-like receptor family pyrin domain containing 3 (NLRP3) inflammasome activity drives the FBR in a nerve injury model yet integration of an NLRP3 inhibitor into the device prevents FBR while allowing full healing of damaged neural tissue to occur.

Implantable electronic medical devices are already widely used for a number of clinical applications, such as pacemakers and cochlear implants. They are also an exciting prospect for the transformative treatment of intractable conditions such as the use of neural electrical stimulators for spinal injury patients. Sustaining long-term implantable device use is limited by the foreign body reaction (FBR) whereby the host recognizes, attacks, and surrounds the device with a dense, fibrotic capsule which, for example, prevents electrical stimulation from reaching the neural interface (1–3). The FBR is driven by the development of an acute inflammatory response against the implant, during which macrophages are recruited to try and phagocytose and then degrade the foreign material, resulting in a process of frustrated phagocytosis (4–6). A chronic inflammatory response is eventually established in which, ultimately, macrophages are thought to coordinate a fibroblast response to surround the implant and form a collagen-rich capsule to separate it from the surrounding tissue. This FBR then persists until the implant is removed from the body (4, 5).

The mechanisms by which the FBR occurs are poorly understood and effective methods to prevent the FBR, without interfering with tissue repair mechanisms, for example after nerve damage, are currently unavailable (7, 8). Different strategies have been tested to prevent the FBR, including local antiinflammatory or antifibrotic agent delivery at the tissue–device interface and/or mechanical matching of the implant and its tissue environment (9–11). None of these approaches have completely succeeded in eliminating the detrimental effects of the FBR. Local delivery of broad-spectrum antiinflammatory drugs, such as dexamethasone, can suppress the FBR, but it prevents neuronal repair (12, 13). There is an urgent need, therefore, to identify novel therapeutic compounds in order to maximize the potential of these transformative medical devices.

One of the key cytokines required for nerve regeneration is interleukin 1β (IL-1β). The messenger RNA (mRNA) for pro-IL-1β is up-regulated by a number of receptors that activate the proinflammatory transcription factor nuclear factor kappa B (NF-κB) (10, 14). Pro-IL-1β is processed to its active form by the inflammasome, a macromolecular signaling platform broadly composed of a receptor nucleotide-binding oligomerization domain-like receptor family pyrin domain containing 3 (NLRP3), an adaptor (apoptosis-associated speck-like protein containing a carboxy-terminal caspase recruitment domain [ASC]), and an effector (caspase-1) (14). FBR complex formation is altered in inflammasome knockout mouse models (15–18) but, because in neuronal injury IL-1β is required for repair (19), it is unclear whether inflammasome inhibitors would improve host tolerance of implantable devices.

The association of frustrated phagocytosis with the pathogenesis of the FBR (6) coupled to the involvement of IL-1β in both inflammation and nerve repair led us to investigate the effects of the NLRP3 inflammasome in two models of the FBR. We first used a murine nerve injury model to characterize the pathogenic changes of the FBR associated with device implantation from the early stages to the chronic fibrotic phase. The innate immune response, particularly that augmented by macrophages and dendritic cells, is the central driver of the FBR. We show that the NLRP3 inflammasome is critical for chronic FBR because local inhibition of this receptor using MCC950 incorporated into the device coating resulted in markedly reduced FBR formation without affecting tissue regeneration. Focal inhibition of NLRP3 also reduced the FBR in a second chronic subcutaneous implantation model. This work thus indicates that local inhibition of NLRP3 could be very effective in preventing the FBR to promote the long-term usage of implantable electronic medical devices, particularly in nervous system applications.

## Results

### RNA Profiling of FBR Tissue Shows Inflammatory and Innate Immune Gene Induction.

**Fig. 1.**
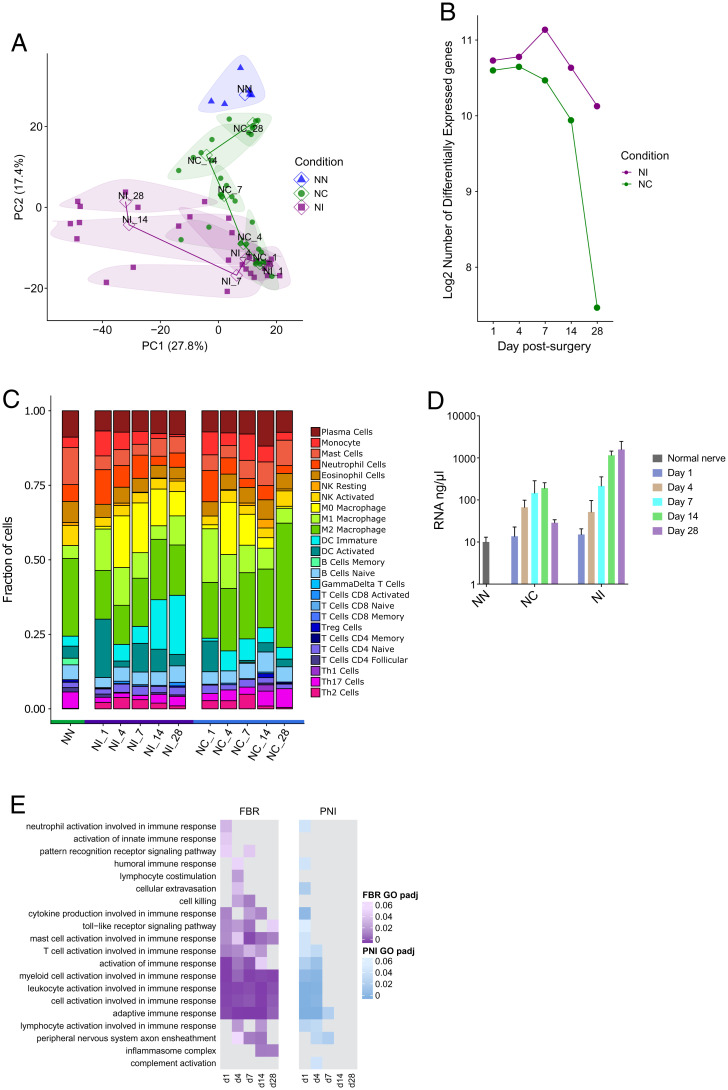
Inflammation is sustained over time in the FBR. RNA-seq results of nerve tissue with an implanted microchannel cuff (NI) and a crushed nerve (NC) at different days postsurgery, compared with a naïve nerve (NN) as a control. Implantation of the microchannel cuff was purposely invasive, requiring surgical dissection of nerve into multiple strands. Samples of NI and NC nerves were analyzed at 1, 4, 7, 14, and 28 d postsurgery. (*A*) PCA of sequencing results from nerve tissue for the top 500 differentially expressed genes. Samples belonging to the same condition and postsurgical day are grouped under the same bubble. Bubbles are also color-coded based on the condition they belong to (NI, purple; NC, green; NN, blue). The number following each group name indicates the days postsurgery. Solid data points correspond to individual operated mice, while the hollow data points represent the average of the group. (*B*) Plot of the number of differentially expressed genes in NI and NC nerves at different time points, relative to NN. (*C*) Breakdown of immune system cell types found in nerves postsurgery, obtained through RNA-seq sample deconvolution. The number following each group name indicates the days postsurgery. (*D*) Plot of the concentration of RNA in sequenced samples. Bars represent the mean; error bars represent SD. DC, dendritic cell; NK, natural killer cell. (*E*) Gene ontology (GO) of immune system processes differentially regulated following device implantation or nerve injury. The adjusted *P* value of pathway change in regulation is represented by changes in color shade in the heatmap (NI, purple; NC, blue), with more significant regulation indicated by darker shades. PNI, peripheral nerve injury.

We implanted microchannel devices into murine peripheral nerves as a clinically important model of the FBR (20). In this model, axons and glia regenerate through the microchannels to restore function and the channels can function as an electrical interface (21, 22). Regeneration is, however, followed by a progressive FBR in which scar tissue extends from the channel walls, eventually compressing and killing the axons, leading to loss of function (23). To identify the potential mechanisms involved in the formation of the FBR, we compared the effects of peripheral sciatic nerve injury (nerve crush; NC) with that of sciatic nerve transection accompanied by implantation of a microchannel device (nerve implant; NI) on gene expression over time (Fig. 1). The NC model used a sciatic nerve crush, leading to axon degeneration distal to the crush followed by rapid regeneration starting within 24 h (24). To make the NI, we fabricated single-channel nerve conduits with a 0.5-mm-diameter microchannel in a 1.5-mm-diameter section of polydimethylsiloxane (PDMS) 3 mm long. The sciatic nerve is cut and this device is inserted between the nerve stumps, and the axons and glia then regenerate through the channels. The FBR fills the channels after 3 mo (23). Previous histological studies show that following implantation of an implant there is an initial acute inflammatory response followed by development of a chronic FBR (4, 23, 25, 26). Samples taken soon after implantation would therefore be expected to show acute changes that are similar in both NCs and nerve conduits but, in later samples, targets unique to the FBR emerge. Successful axon regeneration is demonstrated by sensorimotor testing, observing hindlimb movement and grasping ability (27).

Tissue samples at days 1, 4, 7, 14, and 28 were analyzed by RNA sequencing (RNA-seq). For the first 4 d after surgery, analysis of differentially expressed genes between naïve nerve (NN), NC, and NIs showed large changes in gene expression in both experimental groups relative to NN (Fig. 1*B*). On days 14 and 28, the gene expression pattern of NC returned to normal, but the NI group remained divergent (Fig. 1*B*). These changes in differential expression were accompanied by similar changes in the overall level of RNA extracted from the samples, which provides an approximate estimate of cell number. mRNA levels increased in both experimental groups over the first 7 d, and then decreased in NC (Fig. 1*D*). In the NI group, however, the developing FBR maintained elevated levels of mRNA and therefore cell numbers remained high. In order to see whether the patterns of mRNA expression between the experimental groups diverged from one another, we conducted a principal-component analysis (PCA). This analysis showed that both NC and NI led to similar changes in gene expression immediately following surgery, compared with NN (Fig. 1*A*; NC and NI at days 1, 4, and 7). At later time points, however, and as the nerve injury resolved, the NC gene expression pattern progressively resembled that seen in the NN group. In the NI group, as the acute inflammation transitioned to a chronic inflammatory FBR, a new pattern of gene expression emerged. By 28 d postsurgery, inflammatory gene expression had returned to normal in NC but remained substantially different in the NI group (Fig. 1*A*). We deconvoluted our RNA-seq data to investigate any changes in cell-type populations with the tissues (28). We observed very similar distributions of immune cell types between NI and NC at days 1 and 4 postsurgery, but the pattern diverged between these two groups thereafter (Fig. 1*C*). At day 28, the predominant cell type in NC mice was indicative of predominantly a population of M2 macrophages, consistent with tissue repair after injury (29). In the NI group, however, both proinflammatory M1 and tissue repair M2 macrophages were present and there was also an increase in dendritic cells correlating with both the inflammatory and fibrotic components of the FBR (Fig. 1*C*). This also coincided with an increase in total cell number (as indicated by increased total mRNA content at the latest time point analyzed [28 d]), suggesting that, compared with the NC group, the overall immune cell numbers continued to increase while the FBR was being generated against the implant (Fig. 1*D*). Together, these results suggest that both NC and NI result in acute inflammation followed by tissue regeneration. From 7 d onward, the NC group reverted back to a largely normal gene expression profile, while the NI group demonstrated characteristic, long-lasting changes presumably due to the FBR.

### Inflammasome Genes Are Up-Regulated Only in the NI FBR Tissue.

**Fig. 2.**
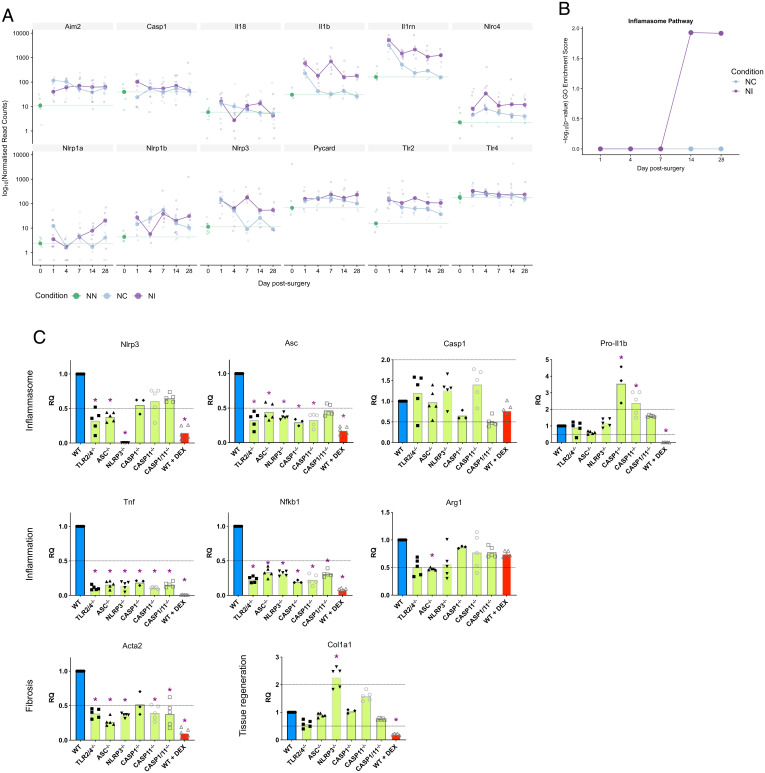
Inflammasome is activated during the FBR. (*A*) Bubbleplot of expression values of various inflammation-related genes, obtained through RNA-seq of mice which had undergone microchannel cuff implantation (NI) or crush nerve injury (NC) or with NN at various days postsurgery. (*B*) Gene ontology of enrichment score of the inflammasome pathway. (*C*) mRNA values in NI capsules of mice implanted with microchannel cuff devices, 28 d postimplantation, analyzed by qPCR. Results are shown for wild-type mice (WT) and knockout models for various inflammasome genes, or wild-type mice with PDMS disks impregnated with the antiinflammatory compound dexamethasone (WT+Dex). Values are presented as mRNA values relative to the wild-type group (RQ). Groups exhibiting a twofold increase or decrease in value (indicated by dashed lines) are identified by a purple asterisk. Bars indicate the group mean; values for individual mice are represented by data points.

To identify whether specific gene pathways were up-regulated in the FBR, we performed a gene ontology analysis of the samples. Gene ontology of differentially regulated immune processes showed that similar pathways were up-regulated in both the NC and NI groups at early time points (1 and 4 d postsurgery), many of which remained up-regulated in NI at later time points (Fig. 1*E*). Genes from the inflammasome complex, however, showed a unique pattern of expression being up-regulated only at the chronic NI time points (14 and 28 d postsurgery) but not in NC. This unique expression profile was validated using qPCR analysis which confirmed that many inflammation-related genes showed increased expression in both the NC and NI groups, but this increase was sustained in the NI group (Fig. 2*A* and *SI Appendix*, Fig. S1). Certain inflammasome genes, such as *Nlrp1a*, had a unique pattern of expression exclusively related to the NI FBR. The gene ontology enrichment score for the inflammasome pathway showed a marked up-regulation of inflammasome-related genes in the chronic time points (days 14 and 28) for the NI group, which were absent in the NC group (Fig. 2*B* and *SI Appendix*, Fig. S1).

### Genes Associated with the FBR, but Not with Tissue Regeneration, Are Suppressed in Inflammasome Knockout Mice.

To determine how the inflammasome may play an important role in the FBR, we performed an NI study in mice lacking functional inflammasome-related (*Asc*, *Nlrp3*, *Casp1*, *Casp11*, *Casp1/11*) or *Tlr2/4* genes (which regulate the expression level of inflammasome components). Gene expression patterns taken from tissues at 28 d postimplantation were analyzed by qPCR and compared between knockout and wild-type mice. We also compared these data with those from wild-type mice implanted with devices which had been impregnated with dexamethasone (NI+Dex), an antiinflammatory drug which disrupts FBR capsule formation and prevents FBR scarring in NIs (9, 12). Antiinflammatory drug impregnation into conduit devices is used to locally deliver anti-FBR treatments both into nerves and in clinically used implants (9, 12). Inflammasome gene expression in the different knockout mice broadly showed decreased mRNA levels, consistent with reduced inflammatory responses (Fig. 2*C*). NI treatment in inflammasome knockout mice led to decreased levels of proinflammatory genes such as *Tnfa* and *Nfkb1* as well as decreased expression of *Acta2* (Fig. 2*C*), a cytoskeletal component up-regulated in myofibroblasts which lay down the fibrotic capsule in the FBR (30). There was, however, a dissociation of the tissue regeneration and fibrosis pathways, with the former preserved in the inflammasome knockout mice following implantation (Fig. 2*C*). The expression of the collagen I gene, for example, which is a primary component of the extracellular matrix produced following nerve injury and, therefore, critical for successful neuronal regeneration, was similar in all animals (Fig. 2*C*). Different patterns of gene expression were seen in the NI+Dex mice. Dexamethasone led to a reduction in the expression of inflammatory markers such as *Tnfa* and *Nfkb1*, as well as a decrease in fibrosis (*Acta2*) (Fig. 2*C*). Despite the effectiveness of dexamethasone in decreasing inflammation and fibrosis following implantation, as has been previously reported (9, 12), the expression of collagen I in dexamethasone-treated mice was also greatly reduced (Fig. 2*C*), pointing toward an impaired tissue regenerative response. These data suggest that the inhibition of the inflammasome could suppress the FBR without compromising tissue repair processes.

### Local Inhibition of NLRP3 Inflammasome Activity by MCC950 Inhibits the FBR but Not Nerve Regeneration.

**Fig. 3.**
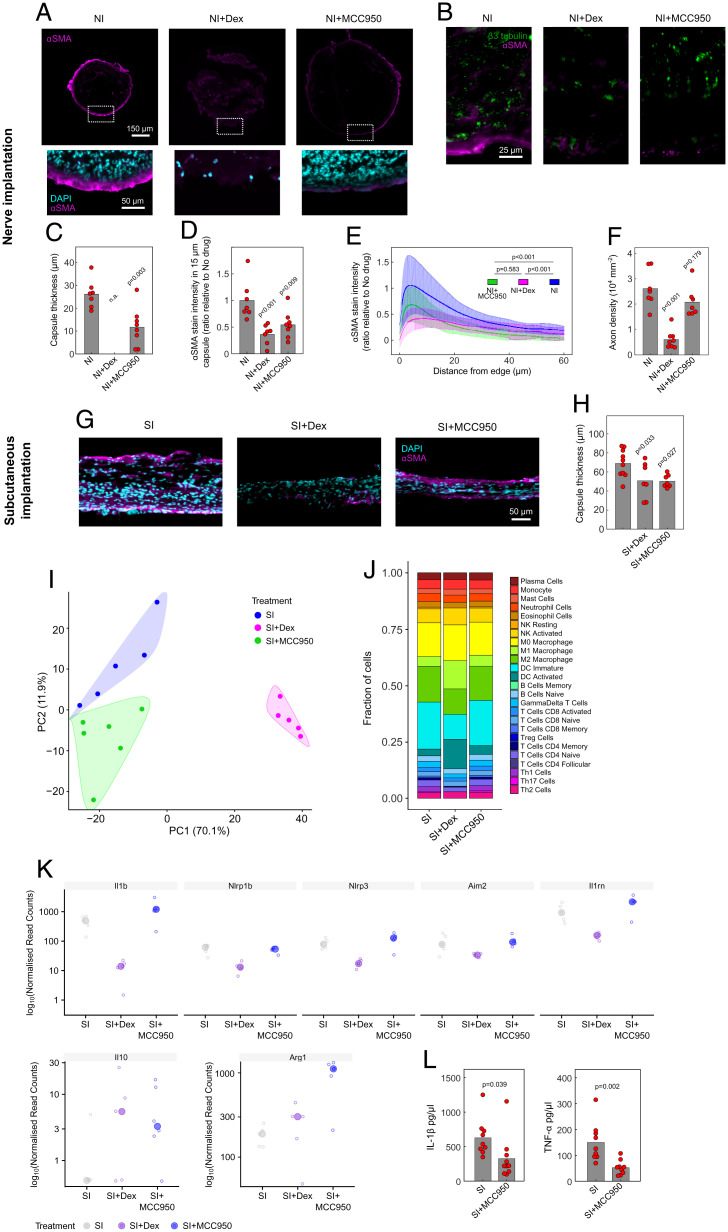
Pharmacological inhibition of NLRP3 significantly reduces the FBR. (*A* and *B*) *Z*-stack confocal images (cross-sections) of the FBR in nerve 3 mo postimplantation of a PDMS conduit. Nerves were transected during implantation to implant the conduit. The PDMS conduit was impregnated with 10 mg/mL of the antiinflammatory drug dexamethasone (NI+Dex), 10 mg/mL of the NLRP3 inhibitor MCC950 (NI+MCC950), or no drug (NI). Tissues were fluorescently labeled for the myofibroblast marker αSMA (magenta), as well as cell nuclei (DAPI; cyan) in *Insets* (*A*), highlighted by white dashed boxes, or for the axon marker β3-tubulin (green) and αSMA (*B*). The FBR is characterized by a ring of myofibroblasts around the edge of the nerve. (*C*) Quantification of FBR capsule thickness around nerves, based on αSMA stain. n.a., not available. (*D* and *E*) Quantification of FBR marker αSMA stain intensity. The plot in *C* consists of the average intensity over the 15 μm closest to the implant edge. (*F*) Quantification of axon density (β3-tubulin stain pattern) in implanted nerves. (*G*) Images of FBR capsules formed around subcutaneously implanted drug-impregnated PDMS disks, 3 mo postimplantation, fluorescently stained for myofibroblasts (αSMA; magenta) and cell nuclei (DAPI; cyan). (*H*) Quantification of FBR capsule thickness in subcutaneous samples (SI). (*I*) PCA of RNA-seq data of nerves 28 d postimplantation in a drug-impregnated PDMS conduit, for the top 500 differentially expressed genes. Samples belonging to the same treatment are grouped under a color-coded bubble. Data points correspond to individual implanted mice. (*J*) Breakdown of immune system cell types found around conduit postimplantation, obtained through RNA-seq sample deconvolution. (*K*) Bubbleplot of expression values of various inflammation-related genes, obtained through RNA-seq. (*L*) ELISA of protein content in subcutaneous disk implants 3 mo postimplantation. Dexamethasone treatment did not produce a sufficiently structured FBR capsule to reliably harvest, and was therefore not included in the analysis. (*C*, *D*, *F*, *H*, and *L*) Circles indicate the average value per mouse, and the gray bar indicates the average of all animals. Statistical comparisons were carried out via one-way ANOVA followed by Dunnett’s multiple-comparisons test comparing groups with the no drug control condition (*D*, *F*, and *H*) or Student’s *t* test (*C* and *L*). (*E*) Solid lines correspond to the average intensity for *n* = 7 or 8 mice at an increasing distance from the implant edge, and the shaded envelope corresponds to the SD. Statistical comparison was done through two-way ANOVA. (*K*) Hollow circles represent normalized read counts per implanted mouse, with the average between all mice per condition shown by a solid circle. All shown genes are statistically significantly different (false discovery rate–adjusted *P* < 0.01).

We hypothesized that NLRP3 was the most likely to be the inflammasome pathway involved in FBR, so we added the NLRP3 selective inhibitor MCC950 (31) to the device coating and tested its effects in our model. We confirmed the efficacy of MCC950 in vitro prior to its use in vivo by showing inhibition of nigericin-induced NLRP3 inflammasome activation in mouse bone marrow–derived macrophages (BMDMs) (*SI Appendix*, Fig. S2). PDMS conduits impregnated with 10 mg/mL MCC950 were implanted onto fully transected sciatic nerves (NI+MCC950). We compared the performance of MCC950-impregnated PDMS conduits with nonimpregnated or PDMS conduits impregnated with dexamethasone (NI+Dex) (Fig. 3*A*). The effect of inhibitors on tissue regeneration and the FBR was analyzed by assessing the degree of axonal regeneration across the conduit and by determining the thickness of the FBR capsule formation, respectively. MCC950-impregnated implants reduced the FBR at the interface between nerve tissue and the PDMS conduit (Fig. 3*C*; capsule thickness at 3 mo postimplantation compared with untreated implants showed a 2.2-fold decrease, *P* = 0.003). The staining intensity for the myofibroblast marker αSMA was also significantly decreased in the NI+MCC950 group, both at the outermost edge of the capsule (Fig. 3*C*; *P* < 0.009) and throughout the entire capsule (Fig. 3*D*; *P* < 0.001), comparable to that seen with NI+Dex. The decrease in the FBR observed with MCC950-containing implants was not seen in NLRP3^−/−^ knockout mice implanted with untreated conduits (*SI Appendix*, Fig. S3), suggesting that a compensatory mechanism develops when NLRP3 is absent at the genetic level. MCC950 did not, however, adversely affect axon regeneration through the conduit implant, as evident by a comparable axon number to native tissue (*P* = 0.179), in contrast to dexamethasone, where the density of axons regrown through the implants 3 mo postimplantation was markedly reduced (Fig. 3 *B* and *F*; *P* < 0.001), as in previous results (12). These data suggest that the anti-FBR effect of locally administered NLRP3 inhibitors results in inhibition of FBR-related inflammation but leaves axonal regrowth unaffected.

We performed RNA-seq of the FBR tissue in nerves implanted with the conduits at 28 d postimplantation. In tissue samples where MCC950 was impregnated into the device, gene expression clustered closer to untreated samples than those treated with dexamethasone (Fig. 3*I*). Sample deconvolution also showed that the immune cell-type populations were very similar in NI and NI+MCC950 (Fig. 3*J*). NI+Dex treatment, in contrast, led to a reduction in a wide range of inflammatory/inflammasome-related genes, including *il1b*, *nlrp3*, and *aim2*, which did not occur in the NI+MCC950 group and also led to changes in the macrophage and dendritic cell populations (Fig. 3*J*). NLRP3 inhibition did lead to some unique changes in gene expression, however, including up-regulation of the M2 macrophage-associated gene *arg1* (Fig. 3*K*).

The effects of NLRP3 inhibition were then tested in a second FBR model. Drug-impregnated PDMS disks were implanted subcutaneously (SIs; subcutaneous implants) in mice (Fig. 3*G*). Three months postimplantation, FBR capsule thickness was significantly reduced to a similar extent in both SI+MCC950 (Fig. 3*H*; *P* = 0.027) and SI+Dex (*P* = 0.033) groups compared with untreated disks, confirming that MCC950 is also an effective anti-FBR treatment in this implantation model. Enzyme-linked immunosorbent assay (ELISA) analysis of the implanted subcutaneous capsules showed that protein levels of the proinflammatory cytokines IL-1β and tumor necrosis factor α (TNF-α) decreased with NLRP3 inhibition (Fig. 3*L*). Collectively, these results suggest that local inhibition of NLRP3 prevents the FBR without suppressing tissue regenerative responses.

## Discussion

Here we show that impregnating implant devices with the NLRP3 inhibitor MCC950 prevents the FBR without affecting tissue regeneration, in contrast to dexamethasone treatment, which prevents the FBR but also blocks axon regeneration (12). Inhibitors of NLRP3 are being developed for a number of clinical applications including inflammatory disease, cancer, sepsis, Alzheimer’s disease, and Parkinson’s disease. They are already being tested in clinical trials for treatment of cryopyrin‐associated periodic autoinflammatory syndromes (32). The potential for local administration of NLRP3 inhibitors has not received much attention, but here we show that, used in this way, these drugs could transform the life of patients with severe injuries or diseases requiring long-term implanted devices by preventing the FBR without affecting tissue regeneration. Currently, there is no similar treatment available and the use of broad-spectrum antiinflammatory drugs like dexamethasone is problematic because it suppresses tissue regeneration, therefore limiting its clinical utility.

The FBR is currently an unavoidable complication of implantation, with the resulting inflammation and fibrosis being among the leading causes of implant failure (1, 2). The detrimental effects of the FBR are widely recognized, yet the cellular and molecular components of this response and how they evolve over time are unclear. Previous studies have shown deposition of a collagen- and αSMA-rich FBR capsule around implants in subcutaneous and nerve implantation models. There has, however, been little characterization of the cell populations involved or how they might change during development of the FBR (33–35).

In the FBR, different cell types are recruited over time, such as macrophages and foreign body giant cells (4). Ultimately, fibroblasts (expressing acta2) are activated by macrophages and mediate fibrosis. This process is very different from tissue regeneration, which is characterized by collagen I deposition. This protein is also laid down by acta2+ fibroblasts and plays a key role in the tissue repair process. Dendritic cells in addition to macrophages have also been implicated in the FBR. They are thought to detect biomaterials via Toll-like receptor 2 (TLR2) and TLR4 to induce pro-IL-1β and TNF-α (36). Over 25% of the monocytes attracted to the site of biomaterial implants mature into dendritic cells and are therefore thought to be important in both the development and resolution of the FBR (37). The exact mechanism and what the involvement of dendritic cells is in the FBR remain poorly understood (38, 39). Here our RNA-seq data suggest that larger numbers of dendritic cells and M1 macrophages but smaller numbers of M2 macrophages are found in the FBR tissue of NI-treated mice when compared with their NC-treated counterparts. Different subsets of macrophages and dendritic cells differ in their proinflammatory capacity, and this is likely to be reflected in the pattern of inflammasome activation in these cellular populations.

Two studies have reported the involvement of inflammasome components in the FBR (15, 17), but without a thorough analysis of the molecular components of the inflammasome throughout the progression of the FBR. In mouse studies, NLRP3, ASC, and caspase-1 were all found to be required for the acute inflammatory response to biomaterials 24 h postimplantation (15), but only ASC and caspase-1 were required for FBR capsule development at 4 wk postimplantation while the role of NLRP3 was dispensable (15). These data suggest that there could be functional redundancy among NLRs, which could explain why we saw no decrease in the FBR in mice lacking NLRP3. Another study reported a reduction in scar thickness at 14 d postimplantation in ASC- and AIM2-knockout mice with only delayed scarring in NLRP3-knockout mice (25). These studies mainly focused on early time points (14 and 28 d) postimplantation, but their findings are collectively in agreement with ours with regard to the phenotype of NLRP3^−/−^ mice. It is increasingly clear that inflammasomes are flexible multiprotein signaling platforms whereby more than one sensor and/or effector caspase can be incorporated to tailor their responses to complex stimuli such as pathogens (16, 40, 41). Myeloid cells use diverse mechanisms to sense particulate and crystalline material (42), which is consistent with the functional redundancy seen in our study. Inflammasome flexibility, therefore, offers the potential for functional redundancy such that when one sensor is absent then another can functionally replace it, which is consistent with our work here and with the earlier AIM2/NLRP3 study (25). Here we saw increased expression of NLRP1a and NLRP1b in our nerve injury models so it is possible this receptor, or another like AIM2, could compensate in driving the FBR when NLRP3 is absent. This further emphasizes the importance of the inflammasome in the FBR, but also supports the notion that there may be redundancy among NLRs driving inflammasome activation. Here we have shown that local inhibition of NLRP3 in wild-type mice is sufficient to markedly reduce the severity of the FBR without adversely affecting neuronal regeneration.

Different approaches have been adopted to minimize the FBR, such as changing the device’s chemical composition or its physical properties or locally delivering antiinflammatory or antifibrotic compounds (10–12, 43). Local drug delivery is an important strategy to control the FBR (44), but the drug choice needs to fit with the local tissue requirements. Different antiinflammatory compounds tested include IL-10 (45), aspirin (15), dexamethasone (12), and cyclosporine A (46) with varying degrees of success but none of them can prevent the FBR without interfering with tissue regeneration. In the context of the peripheral nervous system and particularly when using regenerative neural interface electrodes, it is critical to reduce inflammation and the FBR but not to disturb the process of axonal regeneration. Dexamethasone regulates multiple inflammatory genes, but here we show NLRP3 inhibition in our model specifically targets the expression of antiinflammatory genes such as arginase and IL-10 (Fig. 3*K*). Our work clearly shows that local delivery of MCC950 reduces Il-1β production, chronic inflammation, and scarring, without affecting collagen production and tissue repair. In our earlier work, we showed enhanced IL-1β in a central nervous system FBR model (10), so our work here also has interesting possible therapeutic implications for the management of long-term brain implants, although this requires further investigation.

In conclusion, we have described a therapeutic approach that could markedly improve the potential of implanted electrical devices for sustained use in patients. The coupling of local drug delivery with other approaches such as the use of different device materials and/or softer device coatings could provide a transformative way forward for the long-term use of implantable electronic medical devices.

## Methods

### Device Fabrication.

#### Microchannel cuff devices: NI.

The device was prepared using standard lithography processes. Microchannel arrays were designed in 2D-CAD software (AutoCAD; Autodesk) and the pattern (100 μm wide and tall, 5-mm-long channels with 50-μm-thick walls) transferred to an SU-8 master for the planar microchannel pattern. To improve adhesion, the silicon wafer was first treated with oxygen plasma (30 W, 60 s). SU-8 (GM-1075; Gersteltec) was spun at 2,100 rpm for 45 s to achieve a thickness of 110 μm. The wafer was soft-baked on a hotplate (starting at room temperature, ramped at 2 °C/min to 130 °C, held at 130 °C for 5 min, and ramped back to room temperature at 2 °C/min). The SU-8 was exposed with 1,200 mJ/cm^2^ through a Cr mask. The wafer was then baked on a hotplate (starting at room temperature, ramped at 1.4 °C/min to 100 °C, held at 100 °C for 60 min, and ramped back to room temperature at 0.8 °C/min). The pattern was then developed in propylene glycol methyl ether acetate (PGMEA), rinsed with fresh PGMEA, rinsed with isopropyl alcohol, and dried with nitrogen. To prevent irreversible bonding between the silicon and PDMS, a layer of silane was deposited on the surface of the mold. A layer of trichloro(1H,1H,2H,2H-perfluorooctyl) silane (Sigma-Aldrich; 448931) was transferred to the wafer via vacuum deposition. The mold and a drop of the silane were placed in a vacuum chamber and pumped down for a minimum of 2 h. PDMS was applied over the mold (onto the channel pattern and over a smooth side) and covered with a half (longitudinally cut) rubber tube (Portex; internal diameter 1.4 mm). The PDMS was cured in an oven at 80 °C for 2 h. Once peeled off of the mold, two half cylinders (one with a microchannel pattern and one with the smooth side) were opposed together and sutured with a 9.0 Ethilon suture.

#### Conduit devices: NI.

PDMS was applied over a Petri dish until a 3-mm thickness was achieved. The PDMS was cured in an oven at 80 °C for 2 h. Once peeled off of the mold, the PDMS was first punched with a 1.5-mm punch (World Precision Instruments; 504647) to obtain a cylinder with length 3 mm and diameter 1.5 mm. The cylinder was then punched in the center with a 0.5-mm punch (World Precision Instruments; 504528) to create the inner channel running longitudinal of 0.5 mm in diameter.

#### Subcutaneous discs: SI.

PDMS was applied over a Petri dish until a 1-mm thickness was achieved. The PDMS was cured in an oven at 80 °C for 2 h. Once peeled off of the mold, the PDMS was punched with a 1.5-mm punch (World Precision Instruments; 504647) to obtain a cylinder with thickness 1 mm and diameter 3 mm.

#### Drug impregnation.

Dexamethasone (Sigma-Aldrich; D1881) (12) and the Nlrp3 inhibitor (MCC950) (31) were occasionally added to Sylgard PDMS. Both were added at a concentration of 10 mg of MCC950 or dexamethasone per 1 mL of PDMS.

Implants were stored in phosphate-buffered saline (PBS) and sterilized under ultraviolet light prior to implantation.

### In Vivo Implantation.

All experimental procedures were performed in accordance with UK Animals (Scientific Procedures) Act 1986 or European Parliament and Council Directive 2010/63/EU. Surgical procedures were carried out under aseptic conditions.

All animals used in this study were housed in standard housing conditions with a 12-h light/dark cycle. All animals were female, because they were found to be less prone to autotomy. Where possible, 8- to 12-wk-old female BL6/C57 mice (wild type: Charles River UK; genetically modified: bred in-house) were housed in groups of six per cage and provided ad libitum access to food and water for a minimum of 7 d prior to surgical procedures. Following this period, the sciatic nerve of BL6 mice underwent either a crush injury or was implanted with a device.

Immediately prior to all surgical procedures, animals received an injectable dose of an opioid-based drug. Nonsteroidal antiinflammatory drugs were not used to avoid a confounding factor. Anesthesia for surgical procedures was induced and maintained with isoflurane delivered via a face mask (4% for induction and 1.5 to 2% for maintenance delivered in O_2_ at a 1 to 2 L/min flow). Body temperature was maintained at 37 °C using a thermal pad (monitored with a rectal probe in rats). Animals recovered in a heated environment until fully awake and then returned to their home cage. A further dose of meloxicam was given orally the day after surgery. All work involving live animals complied with University of Cambridge Ethics Committee regulations and was performed under Home Office Project License Number P1E5A5564.

To avoid interoperator variability, every surgical procedure and all implant manufacturing were performed by the same individual with extensive microsurgical training. Additionally, to avoid intraoperator variability, every surgery session consisted of one animal from each experimental group at random order (11 mice per session, half-day). Every surgical session had the assistance and support of the same expert animal facility technician. Normal nerves, used as controls, were explanted using the same system.

#### Sciatic nerve crush.

This procedure was adapted from the literature (47). After preparation, a straight incision over the right thigh was made in the skin. The skin was gently dissected from the underlying musculature. Opening the fascial plane between the gluteus maximus and the anterior head of the biceps femoris revealed the sciatic nerve. The right sciatic nerve was then exposed and gently freed from the surrounding connective tissue using iridectomy scissors. The trifurcation was located and followed 2 mm proximally; this point was used as a landmark for all the crush, transection, or implant procedures. Using a fine 5/45 (Fine Science Tools; 11251-35) forceps, the nerve was placed on the bottom jaw of a superfine hemostatic forceps (Fine Science Tools; 13020-12). The three fascicles were sequentially aligned, not on top of each other. The crush was made perpendicular to the nerve at 45 mm from the third toe, as measured by a thread that approximated the path of the sciatic nerve. The nerve was crushed once for 15 s at three clicks of the hemostatic forceps three times. Care was taken not to stretch the nerve. When the hemostats were reopened, the entire nerve should be translucent at the crush site. The gluteal musculature was reopposed. Finally, the skin incision was closed using 9-mm reflex clips (World Precision Instruments; 500346; applier, 500345).

#### Device implantation.

After preparation, a straight incision over the right thigh was made in the skin. The skin was gently dissected from the underlying musculature. Opening the fascial plane between the gluteus maximus and the anterior head of the biceps femoris revealed the sciatic nerve. The right sciatic nerve was then exposed and gently freed from the surrounding connective tissue using iridectomy scissors. The trifurcation was located and followed 2 mm proximally; this point was used as a landmark for all the crush or implant procedures.

#### Microchannel cuff device.

Using two fine 5/45 (Fine Science Tools; 11251-35) forceps, the nerve was dissected into six to eight bundles of axons and placed in the middle of the “sandwich” microchannel device. The 9/0 suture previously used to secure one side of the device was then knotted on the other side to contain and secure the nerve fascicles inside.

#### Conduit device.

The sciatic nerves were cleanly transected using scissors and the device was positioned between the two resulting nerve stumps. The epineurium of each nerve stump was sutured to the silicone tube using 9/0 nylon sutures (Ethicon). The conduit served as a guide for the regenerating nerve, ensuring reconnection of the two stumps within a few days of recovery. The device was placed in the nerve anatomical compartment and the gluteal musculature was reopposed. Finally, the skin incision was closed using 9-mm reflex clips (World Precision Instruments; 500346; applier, 500345).

#### Subcutaneous discs.

An incision was done dorsally over the right leg of an animal (approximately above the femur). The skin was separated from the underlying muscle fascia using blunt forceps to create a tunnel from the site of incision toward the midline of the animal. The implant was fed through this tunnel and placed just off the midline. Each animal received two subcutaneous implants (same condition): one for ELISA and one for immunohistochemistry.

#### Genetically modified mice.

Wild-type C57BL/6 mice were obtained from Charles River UK. Conventional knockout mice were bred in-house at the animal facilities of the University of Cambridge (Home Office Project License No. 80/2572). *Nlrp3*^−/−^, *Pycard*^−/−^, and *Caspase1/11*^−/−^ mice on a C57BL/6 background were produced by Millennium Pharmaceuticals and obtained from Kate Fitzgerald, University of Massachusetts, Worcester, MA. *Caspase1*^−/−^ and *Caspase11*^−/−^ mice on a C57BL/6 background were provided by Genentech. *TLR2/4*^−/−^ mice on a C57BL/6 background were provided by Shizuo Akira, Osaka University, Osaka, Japan. Mice were backcrossed on a C57BL/6 background at least eight generations. All mice strains were bred independently and routinely genotyped to ensure maintenance of the correct genotype.

### RNA Extraction and Quantification.

RNA extraction was carried out immediately after the animal was killed and the nerve was explanted. Dry ice or liquid nitrogen was used for transport between the animal facility and the laboratory. RNase Zap decontamination solution (Thermo Fisher; AM9780) was used during explantation and extraction to inactivate RNases and prevent sample RNA degradation. The samples were placed in Lysing Matrix D 2-mL tubes (MP Biomedicals; 116913050), where 350 μL of RLT Plus lysis buffer (Qiagen) was added. The Lysing Matrix D 2-mL tubes were inserted into a FastPrep-24 5G instrument, manually set at speed 6.0 m/s for 30 s (two cycles) to disrupt and homogenize the nerves, and then centrifuged for 4 min at 10,000 × *g* at 4 °C to reduce the foam. The samples in buffer were then moved to QIAshredder tubes (Qiagen; 79654) and spun in a microcentrifuge (MSE; Mistral 1000) for 5 min at 12,000 × *g*. This step served not only to further homogenize the samples as part of the RNA extraction protocol but also to filter out the beads from the Lysing Matrix D 2-mL tubes. The RNeasy Plus Micro Kit (Qiagen; 74034) was used for RNA extraction in all the experiments and according to the manufacturer’s instructions to obtain RNA diluted in 12 μL of RNase-free water. The RNA-containing flow-through was collected and stored at −80 °C.

RNA quantification and integrity analysis were carried out on all samples prior to library preparation and qRT-PCR; 1.5 μL of each RNA sample was aliquoted and allocated for this purpose (0.5 μL excess to account for any pipetting error and residual) using RNA 6000 Pico or Nano kits (Agilent; 5067-1513 and 5067-1511), depending on the expected yield. Samples were thawed and denatured through a 2-min incubation step at 70 °C, followed by rapid cooling in ice. An RNA Pico or Nano chip was loaded with the running gel and dye (Agilent), and 1 μL of the denatured RNA sample was added. The chip was then transferred to an Agilent 2100 Bioanalyzer System for analysis. The resulting RNA profile was analyzed in 2100 Expert software (Agilent). The concentration and the integrity of the extracted total RNA were quantified. All samples were classified according to their degradation state (RNA integrity number; RIN). Only samples with a RIN above 3 were processed.

### RNA-Seq.

#### Experimental plans.

##### *Fig. 1*.

There were 11 groups, with 3 conditions (naïve nerve, NN; injured nerve, NC; nerve implanted with microchannel cuff devices, NI), with explantation at different time points postsurgery (days 1, 4, 7, 14, and 28): NN; NC day 1, NC day 4, NC day 7, NC day 14, and NC day 28; and NI day 1, NI day 4, NI day 7, NI day 14, and NI day 28.

##### *Fig. 3.*

There were 3 groups, with 3 conditions (nerve implanted with untreated conduit device, no drug; nerve implanted with conduit device impregnated with dexamethasone, Dex; nerve implanted with conduit device impregnated with MCC950, NLRP3inh), with explantation at day 28 postsurgery.

#### Complementary DNA library preparation.

Two different kits were used for complementary DNA preparation according to manufacturer instructions: Ovation RNA-Seq System V2 Kit (NuGen; 7102-32) for Fig. 1; and SMARTer Stranded Total RNA-Seq Kit, Pico Input Mammalian (Takara Bio USA; 063017) for Fig. 3.

#### Sequencing.

After each experiment, the libraries from all the samples were normalized and pooled together into a single aliquot which was submitted for sequencing using Illumina technology at the Cancer Research UK genomic core facility.

#### Processing of RNA-seq.

Data were aligned to the mm10 mouse genome (Ensembl Release GRCm38.p5) with STAR (v020201) (48). Alignments and quality control (QC) were processed using Cluster Flow (v0.5dev) (49) pipelines (FASTQC, Trim_galore) and summarized using MultiQC (0.9.dev0) (50). Gene quantification was determined with HTSeq-Counts (v0.6.1p1) (51). Additional QC was performed with featureCounts (v1.5.0-p2) (52) and Qualimap (v2.2) (53). Differential gene expression was performed with the DESeq2 package (v1.18.1, R v3.4.0) (54) and, with the same package, read counts were normalized on the estimated size factors. Technical replicates run on separate lanes were collapsed using DESeq2. UpSetR is an alternative for plotting sets of data to visualize overlaps as a more intuitive replacement for Euler/Venn diagrams (55).

#### Deconvolving RNA-seq.

Samples were deconvolved for fractions of the immune cell types present, using DeconRNASeq (28) and a signature matrix that distinguishes 25 mouse hematopoietic cell types, including six major cell types: granulocytes, B cells, T cells, natural killer cells, dendritic cells, and mono/macrophages (56).

#### Software.

Software used is listed in *SI Appendix*, Table S1. Scripts to analyze the RNA-seq data are available at https://github.com/CTR-BFX/CTR_kf284_0002. RNA-seq data have been deposited in the ArrayExpress database at the European Molecular Biology Laboratory’s European Bioinformatics Institute (EMBL-EBI) under accession numbers E-MTAB-10293 and E-MTAB-10294.

#### Data interpretation.

Fold changes and *P* values were calculated for each gene among each group. Standard QC steps (PCA and hierarchical clustering) were run together with data visualization techniques (volcano plots, MA plots) as part of the analysis pipeline. Genes expressed were filtered to produce lists of differentially expressed genes. These were defined as genes with a minimum of a twofold change in expression, a base expression above three normalized counts, and an adjusted *P* value below 0.01.

### qRT-PCR.

The iTaq Universal SYBR Green One-Step Kit (Bio-Rad; 172-5150) was used according to the manufacturer’s instructions. Commercial validated primers were used (Bio-Rad; PrimePCR).

#### Result analysis and interpretation.

Relative expression of genes of interest was measured using the RQ (relative quantification) method. RQ was considered significant when there was a minimum of a twofold change: RQ of more than 2 or less than 0.5 (57). Housekeeping genes (or endogenous control: a gene that does not vary between all of the samples tested) were selected from the literature (58, 59) and validated using the RNA-seq dataset from Fig. 1.

### In Vitro Characterization of the Efficacy of MCC950.

MCC950 was used in in vitro and in vivo experiments (31). Inflammasome-derived cytotoxicity and IL-1β suppressive effects were validated using immortalized BMDMs. Cells were cultured in Dulbecco’s modified Eagle’s complete medium (Sigma), seeded in 96-well flat-bottom plates at 2 × 10^5^ cells per well, and incubated overnight at 37 °C with 5% CO_2_. The following day, cells were primed for 3 h with lipopolysaccharide (LPS) at 200 ng/mL. After priming, cells were treated for 1 h with MCC950 (final concentration 10 μM) and another hour with 200 μL nigericin at 10 and 20 μM final concentrations (both incubations were at 37 °C with 5% CO_2_). After incubation, cell death (lactate dehydrogenase [LDH] release) was measured from the supernatant using the CytoTox 96 Non-Radioactive Cytotoxicity Assay (Promega; G1780), following the manufacturer’s instructions. Three replicates were used for each condition. LDH and LPS + nigericin (10 and 20 μM final concentrations) were used as positive controls (provided by the manufacturer). Media only, LPS only, and MCC6642 (a nonfunctioning replicate of MCC950) were used as negative controls. LPS + nigericin + MCC950 was used to test MCC950 cytotoxic activity.

### ELISA.

Secreted cytokines were measured in the culture supernatants of the BMDMs treated per an MCC950 cytotoxicity assay or in the extracellular space of fresh tissue (capsule around subcutaneous discs) explanted from mice. All cytokines were measured according to the manufacturer’s instructions. For IL-1β, the OptEIA Mouse IL-1β Set (BD Biosciences) was used. For TNF-α, the DuoSet ELISA Kit (R&D Systems) was used. ELISA was not conducted on nerve tissue due to an insufficient amount of tissue harvested in this implantation model.

#### Result analysis and interpretation.

The experimental results were analyzed using a one-way ANOVA with Tukey’s or *t* test multiple-comparisons test.

### Immunohistochemistry.

All tissue was fixed prior to processing and staining by immersion in paraformaldehyde solution (40 mg/mL in PBS) overnight at 4 °C. Samples which required sectioning were then transferred to a sucrose solution (30% [weight/weight] in PBS; S0389; Sigma-Aldrich) for cryoprotection. They were kept in this solution for a minimum of 16 h at 4 °C, and otherwise stored until further processing. Cryopreserved samples were embedded in optimal cutting temperature compound (Tissue-Tek; 4583), which was frozen and mounted on a cryostat (CM3050 S; Leica). Sections (12 μm thick) were cut from the samples at a cutting temperature of −20 °C. Sections were placed on glass slides and allowed to dry at room temperature overnight before storage at −20 °C until stained.

Sections ready to be stained were washed in a Triton X-100 0.1% (volume/volume; vol/vol) solution in PBS to allow for permeabilization. These and all further washes were performed three times for 10 min. To minimize nonspecific antibody binding, sections were incubated in a blocking buffer consisting of Tris-buffered saline containing 0.03% (vol/vol) Triton X-100 and 10% (vol/vol) donkey serum (Millipore; S30-100ML). After blocking for 1 h at room temperature, primary antibodies (in 10% blocking buffer [vol/vol] in PBS) were added to the sections (*SI Appendix*, Table S2). Sections were covered with paraffin film to prevent drying and were incubated in primary antibodies overnight at 4 °C.

Sections were washed in PBS/Triton solution to remove excess primary antibodies, and then incubated in secondary antibodies in the same solution as for the primary antibodies for 2 h at room temperature. Secondary antibodies were finally washed off with two PBS washes followed by a nonsaline Tris-buffered solution (T6066; Sigma-Aldrich). FluorSave mounting agent (Millipore; 345789) was added to sections to preserve fluorescence before encasing with a glass coverslip and storing at 4 °C prior to imaging.

Imaging of stained nerve tissue was carried out using a confocal microscope (Leica; TCS SP5). Image files were exported and processed for analysis in the ImageJ software package (v1.48; NIH). Stain intensity profiles of FBR capsules was carried out through a combination of custom Matlab and Fiji scripts. The edge of the nerve capsule was delineated by the user and aligned by the scripts. An intensity profile (intensity vs. depth into the nerve) of each stain was obtained. The average intensity from the edge of the nerve to a depth of 25 μm was calculated and provided as a ratio to the same intensity of the NI group. The only exception was CD68 stains, where a depth of 50 μm was instead chosen, as macrophages were found to mostly locate deeper into the tissue than other markers. Capsule thickness was analyzed using a Matlab script, after its edge was marked by hand based on the αSMA stain. Axon density was analyzed in an automated fashion using a Fiji script over three randomly chosen 100 × 100-μm boxes for every image. This was done 5 mm distal to the point of conduit implantation to minimize any effects from the conduits on regeneration, as this could result in a nonuniform axon distribution. Statistical analysis and data plotting were carried out using MATLAB (MathWorks; R2016b).

### Data Quantification, Graph Plotting, and Statistical Analysis.

All graph styles and statistical analysis tests were specifically selected for each type of experiment to provide as much relevant information as possible and carry out the most powerful statistical comparisons given the type of data. Wherever possible, plots present all data points gathered from each condition.

Statistical comparison was carried out using parametric ANOVA tests. Parametric Student’s *t* tests or nonparametric Mann–Whitney *U* tests were instead used in cases of two sample comparisons. Parametric post hoc comparisons consisted of Tukey’s test or, where a clear control group could be identified, Dunnett’s test. The choice of parametric or nonparametric statistical comparisons was taken based on histogram plots.

All plotting and statistical analysis (excluding RNA-seq analysis) were carried out in Prism 8 (GraphPad Software) or MATLAB.

## Supplementary Material

Supplementary File

## Data Availability

The RNA-seq data reported in this article have been deposited in ArrayExpress, EMBL-EBI (accession nos. E-MTAB-10293 and E-MTAB-10294). Scripts to analyze the RNA-seq data are available at https://github.com/CTR-BFX/CTR_kf284_0002. All study data are included in the article and/or *SI Appendix*.
