# Supplementary material for: Prevention of the foreign body response to implantable medical devices by inflammasome inhibition
Source: Proc Natl Acad Sci U S A. 2022 Mar 17;119(12):e2115857119. doi: 10.1073/pnas.2115857119 (PMC8944905; doi:10.1073/pnas.2115857119)
Supplement: Supplementary File [file pnas.2115857119.sapp.pdf]

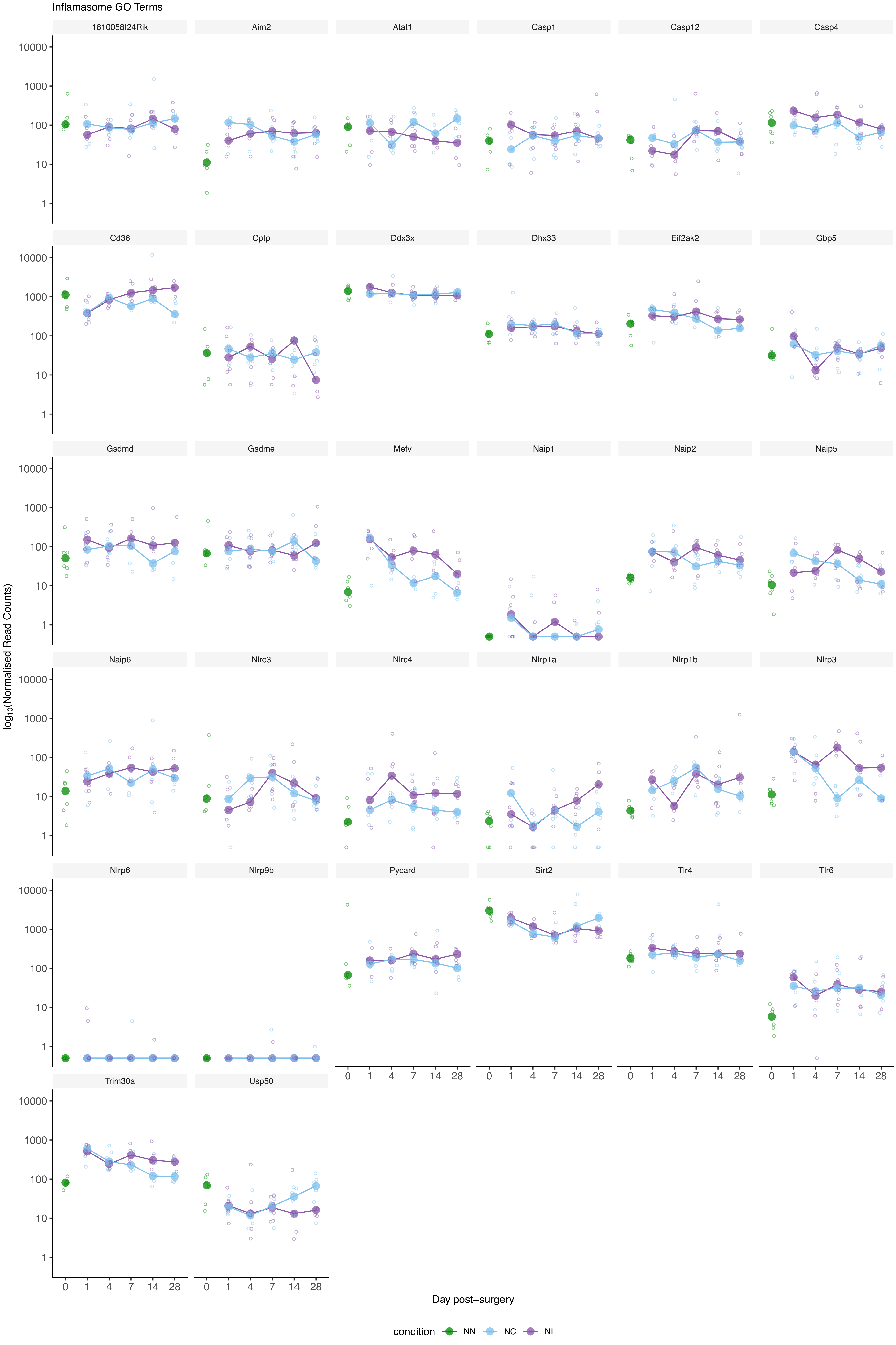

Inflamasome GO Terms
1810058I24Rik
Aim2
Atat1
Casp1
Casp12
Casp4
Cd36
Cptp
Ddx3x
Dhx33
Eif2ak2
Gbp5
Gsdmd
Gsdme
Mefv
Naip1
Naip2
Naip5
Naip6
Nlrc3
Nlrc4
Nlrp1a
Nlrp1b
Nlrp3
Nlrp6
Nlrp9b
Pycard
Sirt2
Tlr4
Tlr6
Trim30a
Usp50
log10(Normalised Read Counts)
10000
1000
100
10
1
0 1 4 7 14 28
Day post-surgery
condition
NN
NC
NI

Supplementary Figure 1. Bubbleplot of expression values of all genes in the the inflammasome complex gene ontology term (GO:0061702), as well as Gasdermin E (Gsdme). Obtained through RNA sequencing of mice which had undergone microchannel cuff implantation (NI), crush nerve injury (NC), or naïve nerves (NN), at various days post-surgery. A subset of these plots are shown in Fig. 2e. Their combined expression is shown under inflammasome complex gene ontology (Fig. 1e, Fig. 2b).

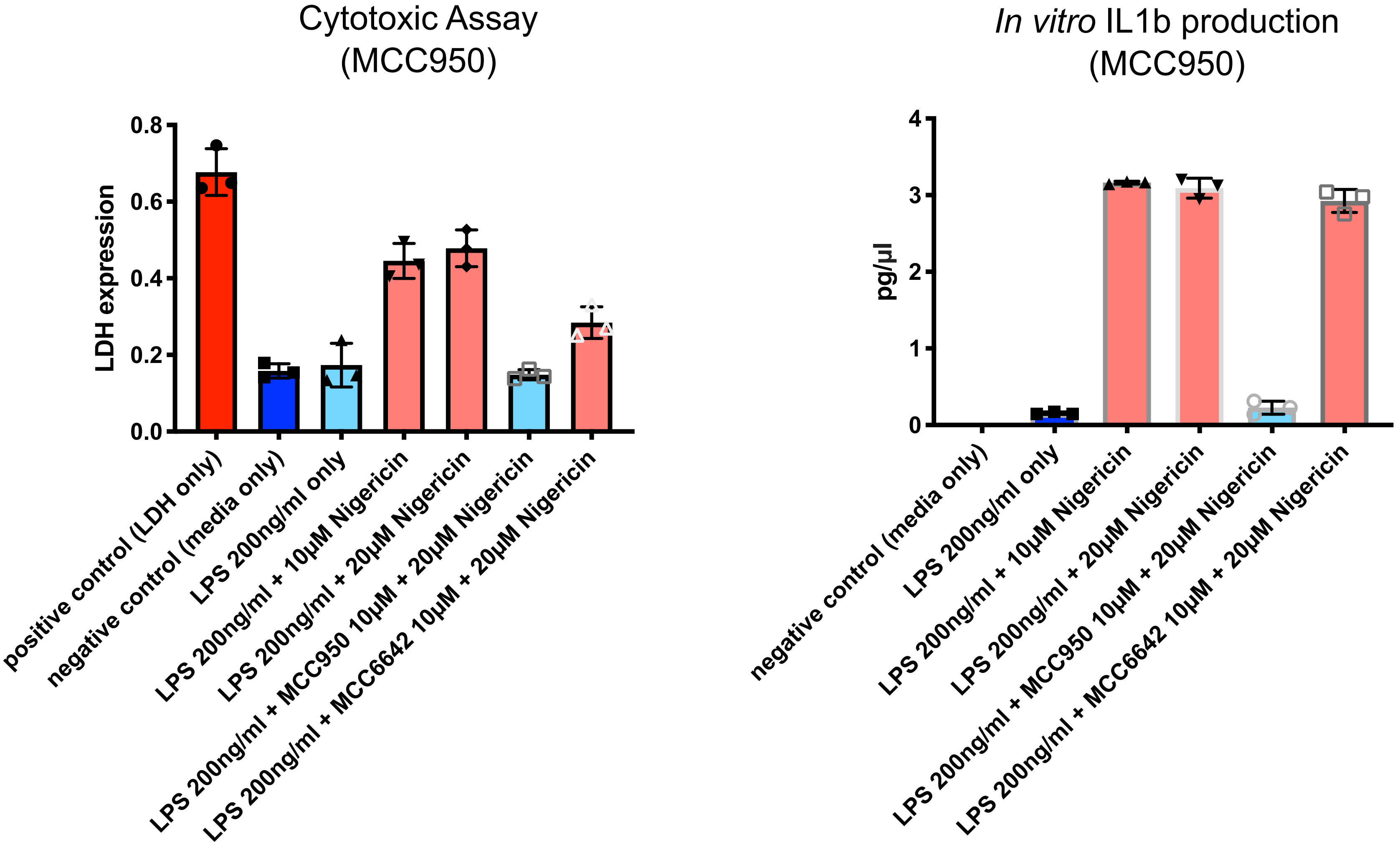

Cytotoxic Assay
(MCC950)
LDH expression
0.0
0.2
0.4
0.6
0.8
positive control (LDH only)
negative control (media only)
LPS 200ng/ml only
LPS 200ng/ml + 10µM Nigericin
LPS 200ng/ml + 20µM Nigericin
LPS 200ng/ml + MCC950 10µM + 20µM Nigericin
LPS 200ng/ml + MCC6642 10µM + 20µM Nigericin
In vitro IL1b production
(MCC950)
pg/µl
0
1
2
3
4
negative control (media only)
LPS 200ng/ml only
LPS 200ng/ml + 10µM Nigericin
LPS 200ng/ml + 20µM Nigericin
LPS 200ng/ml + MCC950 10µM + 20µM Nigericin
LPS 200ng/ml + MCC6642 10µM + 20µM Nigericin

Supplementary Figure 2. Validation of MCC950 inhibiting NLRP3. MCC950 inhibits the production of the inflammasome product IL-1b *in vitro* (right), analyzed by ELISA. The inactive form MCC6642 used as a control does not affect its production. MCC950 does not produce any cytotoxicity *in vitro* (left), as characterized by LDH expression in a cytotoxic assay. Bar indicates sample mean, with bars indicating standard deviation. *In vitro* replicates represented as data points.

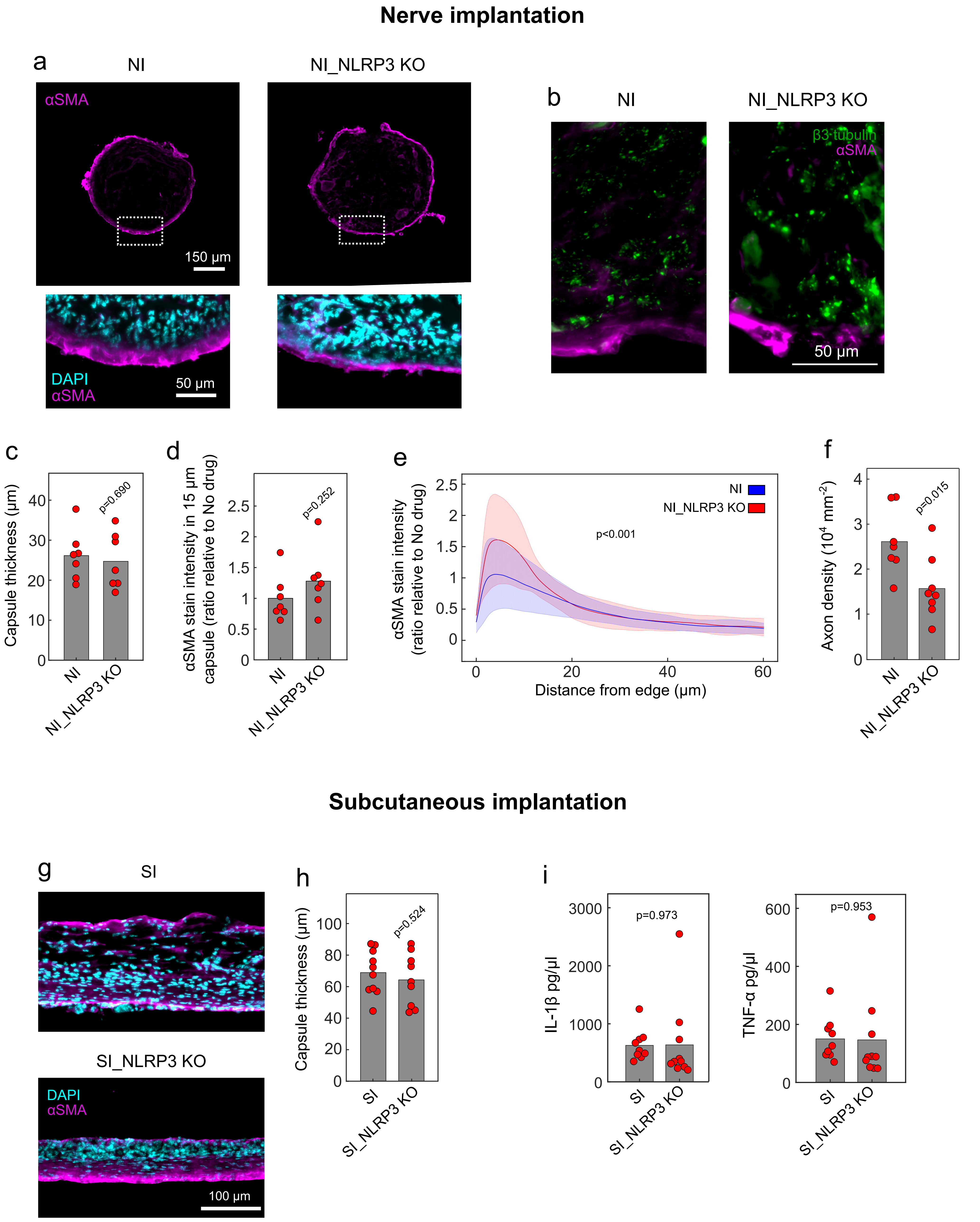
Nerve implantation
a
NI
NI_NLRP3 KO
αSMA
150 µm
DAPI
αSMA
50 µm
b
NI
NI_NLRP3 KO
β3-tubulin
αSMA
50 µm
c
Capsule thickness (µm)
p=0.690
NI
NI_NLRP3 KO
d
αSMA stain intensity in 15 µm capsule (ratio relative to No drug)
p=0.252
NI
NI_NLRP3 KO
e
αSMA stain intensity (ratio relative to No drug)
NI
NI_NLRP3 KO
p<0.001
Distance from edge (µm)
f
Axon density (10^4 mm^-2)
p=0.015
NI
NI_NLRP3 KO
Subcutaneous implantation
g
SI
SI_NLRP3 KO
DAPI
αSMA
100 µm
h
Capsule thickness (µm)
p=0.524
SI
SI_NLRP3 KO
i
IL-1β pg/µl
p=0.973
SI
SI_NLRP3 KO
TNF-α pg/µl
p=0.953
SI
SI_NLRP3 KO

Supplementary Figure 3. NLRP3 knockout mice develop normal levels of FBR. **a-b**, Z-stack confocal images (cross-sections) of FBR in nerve 3 months post-implantation of PDMS conduit. Conduits were implanted into wild type (NI) or into NLRP3$^{-/-}$ (NI_NLRP3 KO) mice. The tissue was fluorescently labelled for the myofibroblast marker αSMA (magenta), as well as cell nuclei (DAPI, cyan) in the insets (a); or for axon marker β3 tubulin (green) and αSMA (b). Inset highlighted by white dashed box. FBR to the conduit is characterized by a ring of myofibroblasts around the edge of the nerve **c**, Quantification of FBR capsule thickness around nerves, based on αSMA stain. **d-e**, Quantification of FBR marker αSMA stain intensity. Plot in c consists of average intensity over the 15 µm closest to the implant edge. **f**, Quantification of axon density (β3 tubulin stain pattern) in implanted nerves. **g**, Images of FBR capsules formed around subcutaneously implanted PDMS disks (SI), 3 months post-implantation, fluorescently stained for myofibroblasts (αSMA, magenta) and cell nuclei (DAPI, cyan). **i**, ELISA of protein content in subcutaneous disk implants. For plots c-d, f, h, and i: circles indicate average value per mouse, and grey bar the average of all animals. Statistical comparisons carried out via Student’s t-test. For plot e: solid lines correspond to average intensity for N = 7 mice at an increasing distance from implant edge, and the shaded envelope corresponds to the standard deviation. Statistical comparison done through two-way ANOVA.

Resource	URL
GRCm38	http://mar2016.archive.ensembl.org/index.html
FastQC	http://www.bioinformatics.babraham.ac.uk/projects/fastqc/
Trim_galore	http://www.bioinformatics.babraham.ac.uk/projects/trim_galore/
STAR	http://dx.doi.org/10.1093/bioinformatics/bts635
HTSeq-counts	http://dx.doi.org/10.1093/bioinformatics/btu638
Feature_counts	http://dx.doi.org/10.1093/bioinformatics/btt656
Qualimap	http://dx.doi.org/10.1093/bioinformatics/bts503
ClusterFlow	http://dx.doi.org/10.12688/f1000research.10335.2
MultiQC	http://dx.doi.org/10.1093/bioinformatics/btw354
DESeq2	http://dx.doi.org/10.1186/s13059-014-0550-8
UpSetR	http://dx.doi.org/10.1093/bioinformatics/btx364
DeconRNASeq	http://dx.doi.org/10.1093/bioinformatics/btt090
Immune cell Reference	http://dx.doi.org/10.1038/srep40508

Supplementary Table 1. Software packages used in RNA sequencing data analysis.

Antibody	Dilution	Source	Code
**Primary antibodies**			
Rabbit anti-beta Tubulin	1:200	Abcam	ab6046
Mouse anti-αSMA	1:100	Abcam	ab7817
**Secondary antibodies**			
Alexa Fluor donkey anti-mouse 555	1:1,000	Invitrogen	ab150106
Alexa Fluor donkey anti-rabbit 488	1:1,000	Invitrogen	ab150073
Hoechst-33342 nuclear stain	1:10,000	Sigma	14533

Supplementary Table 2. Antibodies used in immunohistochemistry experiments.
